# *Acinetobacter baumannii* from Samples of Commercially Reared Turkeys: Genomic Relationships, Antimicrobial and Biocide Susceptibility

**DOI:** 10.3390/microorganisms11030759

**Published:** 2023-03-16

**Authors:** Anna Schmitz, Dennis Hanke, Dörte Lüschow, Stefan Schwarz, Paul G. Higgins, Andrea T. Feßler

**Affiliations:** 1 Institute of Poultry Diseases, School of Veterinary Medicine, Freie Universität Berlin, 14163 Berlin, Germany; 2 Veterinary Centre for Resistance Research (TZR), Freie Universität Berlin, 14163 Berlin, Germany; 3 Institute of Microbiology and Epizootics, Centre for Infection Medicine, School of Veterinary Medicine, Freie Universität Berlin, 14163 Berlin, Germany; 4 Institute for Medical Microbiology, Immunology and Hygiene, Faculty of Medicine and University Hospital Cologne, University of Cologne, 50935 Cologne, Germany; paul.higgins@uni-koeln.de; 5 German Center for Infection Research (DZIF), Partner Site Bonn-Cologne, 50935 Cologne, Germany; 6 Center for Molecular Medicine Cologne, Faculty of Medicine and University Hospital Cologne, University of Cologne, 50935 Cologne, Germany

**Keywords:** poultry, antimicrobial resistance, biocide resistance, PFGE, WGS, Pasteur MLST scheme, Oxford MLST scheme, core genome MLST

## Abstract

*Acinetobacter baumannii* is especially known as a cause of nosocomial infections worldwide. It shows intrinsic and acquired resistances to numerous antimicrobial agents, which can render the treatment difficult. In contrast to the situation in human medicine, there are only few studies focusing on *A. baumannii* among livestock. In this study, we have examined 643 samples from turkeys reared for meat production, including 250 environmental and 393 diagnostic samples, for the presence of *A. baumannii.* In total, 99 isolates were identified, confirmed to species level via MALDI-TOF-MS and characterised with pulsed-field gel electrophoresis. Antimicrobial and biocide susceptibility was tested by broth microdilution methods. Based on the results, 26 representative isolates were selected and subjected to whole-genome sequencing (WGS). In general, *A. baumannii* was detected at a very low prevalence, except for a high prevalence of 79.7% in chick-box-papers (n = 118) of one-day-old turkey chicks. The distributions of the minimal inhibitory concentration values were unimodal for the four biocides and for most of the antimicrobial agents tested. WGS revealed 16 Pasteur and 18 Oxford sequence types, including new ones. Core genome MLST highlighted the diversity of most isolates. In conclusion, the isolates detected were highly diverse and still susceptible to many antimicrobial agents.

## 1. Introduction

*Acinetobacter baumannii* are nonmotile, oxidase-negative, aerobic, Gram-negative coccobacilli [1]. These bacteria are associated with nosocomial infections worldwide [2]. Although *A. baumannii* is an opportunistic pathogen, it has led to many outbreaks in hospitals and care-facilities with high morbidity and mortality rates [3]. These infections are mainly caused by outbreak strains, which can spread rapidly between patients [4]. Many disease conditions, including ventilator-associated pneumonia, bloodstream infection, urinary tract infection, wound infection and meningitis have been described [3], and *A. baumannii* has been shown to be a common co-infecting agent in COVID-19 patients in intensive care units [5,6]. *A. baumannii* can rapidly develop antimicrobial resistance [7,8] due to various resistance mechanisms, such as β-lactamase production, efflux pump overexpression, alterations at the target sites of the antimicrobial agents, and decreased membrane permeability [9]. *A. baumanni* is intrinsically resistant to a number of antimicrobial agents, such as penicillin, ampicillin, amoxicillin, amoxicillin-clavulanic acid, aztreonam, first generation cephalosporins (cephalothin, cefazolin), second generation cephalosporins (cefuroxime), cephamycines (cefoxitin, cefotetan), clindamycin, daptomycin, fusidic acid, glycopeptides (vancomycin), linezolid, macrolides (erythromycin, azithromycin, clarithromycin), quinupristin-dalfopristin, rifampin, ertapenem, trimethoprim, chloramphenicol, and fosfomycin [10]. Moreover, multi-drug resistance properties include resistance not only to the most commonly used antimicrobial agents, but also to last-resort antimicrobial agents in human medicine [11,12]. Due to its outstanding ability to escape antimicrobial therapy, *A. baumannii* is listed among the ESKAPE pathogens, which also include *Enterococcus faecium*, *Staphylococcus aureus*, *Klebsiella pneumoniae*, *Pseudomonas aeruginosa,* and *Enterobacter* spp. [13,14].

Concerning farm animal populations and their environment, there is little information about the distribution of *A. baumannii*, with data on antimicrobial resistance especially lacking [15]. A recent study on isolates from cattle has shown that they harbour a highly diverse population of *A. baumannii*, which are susceptible to most antimicrobial agents [16].

Concerning poultry, in 2011 a case was described in which a highly virulent strain of *A. baumannii* led to an outbreak in a commercial chicken farm in China, during which more than 3000 six-day-old chicks died [17]. Otherwise, the isolation of *A. baumannii* has only occasionally been described from chickens and was not related to outbreaks or diseases [18,19]. Among other bird species, in Poland, 25% of 661 white stork (*Ciconia ciconia*) nestlings were tested positive for *A. baumannii* [18]. There are also reports of single *A. baumannii* isolates found in geese [18], falcons [20], and other birds of which the species was not published [21,22,23]. However, in general, birds are not considered as a primary host for *A. baumannii* [24].

In environmental samples associated with poultry, *A. baumannii* isolates have been obtained from sewage water of a poultry slaughterhouse [25]. It has also been detected in the air of a duck hatchery. The authors considered that these bacteria might be a possible trigger for respiratory diseases in hatchery workers [26,27]. Liu et al. also point out that cross-infections between humans and chicks through handling may be possible [17]. Therefore, the dissemination of *A. baumannii* in poultry livestock may have far-reaching consequences for public health [18]. In addition, poultry meat might potentially be a threat to public health, as *A. baumannii* has been isolated from raw turkey and chicken meat [28,29,30,31,32].

In our pilot study, we focused on the occurrence of *A. baumannii* in samples from commercially reared turkeys for meat production, as information concerning these farm animals and especially their antimicrobial resistance profiles are missing [15]. Collected isolates were characterised by pulsed-field gel electrophoresis and whole-genome sequencing, and tested for antimicrobial and biocide susceptibility.

## 2. Materials and Methods

### 2.1. Sample Collection and Isolation

In total, 250 samples from 95 different farms were collected from allegedly healthy commercial fattening turkey flocks distributed all over Germany (n = 94) and the Czech Republic (n = 1) as part of a *Salmonella* surveillance in 2019. This included 118 chick-box-papers (paper with wood shavings on which the turkey chicks were transported from the hatchery to the production house containing meconium) from one-day-old turkey chicks taken on arrival at the production house from 81 farms (with 24 farms providing more than one sample). Six unused chick-box-papers were also examined as negative controls. In addition, 50 boot swab samples (containing one pair of boot swabs each) taken during the rearing period and 82 boot swab samples from turkeys leaving for the slaughterhouse were investigated. Data and subsequent results were compiled, assessed, and evaluated using Microsoft Excel (Microsoft Office 2019). After pre-enrichment in buffered peptone water (Thermo Scientific, Wesel, Germany) at 37 °C for 16 to 18 h, approximately 10 μL enrichment broth was streaked on chromogenic media Brilliance UTI Clarity agar (Thermo Scientific, Wesel, Germany) and incubated at 37 °C for 24 h. Buffered peptone water without any supplements was analysed as sterility control. In addition, 393 diagnostic samples sent to the Institute of Poultry Diseases, Freie Universität Berlin, Berlin, Germany between 2018–2020 were examined. These included liver and yolk sac samples from 88 one-to six-day-old commercial turkey chicks, as well as 217 lung- and heart-swabs from commercial turkeys. Cultivation was performed on Columbia agar with 5% sheep blood (Thermo Scientific, Wesel, Germany) and Brilliance UTI Clarity agar at 37 °C for 24 h.

Presumptive colonies were selected, sub-cultured, and confirmed to species level by matrix-assisted laser desorption/ionisation time of flight mass spectrometry (MALDI-TOF MS) (Bruker Daltonic GmbH, Bremen, Germany). All isolates were stored at −20 °C in brain heart infusion (BHI) medium (Roth, Karlsruhe, Germany) until further use.

### 2.2. Antimicrobial Susceptibility Testing

Antimicrobial susceptibility testing was performed by broth microdilution according to the instructions of the Clinical and Laboratory Standards Institute (CLSI, 2022) [10]. The *Acinetobacter* isolates were tested with custom-made microtiter plates (MCS Diagnostics, Swalmen, The Netherlands) for their susceptibility to 18 antimicrobial agents or combinations: colistin, streptomycin, neomycin, trimethoprim/sulfamethoxazole, gentamicin, nalidixic acid, ciprofloxacin, enrofloxacin, marbofloxacin, tetracycline, doxycycline, florfenicol, imipenem, ceftiofur, cefquinome, cefotaxime, cefoperazone, and tiamulin. This test panel was the same as used in the GE*RM*-Vet programme, the German national resistance monitoring programme of veterinary pathogens, for Gram-negative bacteria. The reference strain *Escherichia coli* ATCC^®^ 25922 served as quality control. The minimal inhibitory concentration (MIC) values were interpreted as susceptible, intermediate, or resistant using the human-specific clinical breakpoints from CLSI [10], as veterinary-specific clinical breakpoints are not available for *Acinetobacter* spp.

### 2.3. Biocide Susceptibility Testing

Biocide susceptibility testing was performed for four different biocides—benzalkonium chloride (a quaternary ammonium compound), octenidine (a bispyridine) as well as chlorhexidine and polyhexanide (two biguanides)—using commercial microtitre plates (sifin diagnostics GmbH, Berlin, Germany) and the protocol from Schug et al. [33], with some adaptations [34]. The use of commercial microtitre plates led to an adaptation of the protocol by adding only 30 µL bacterial suspension of a density of 0.5 McFarland to 12 mL single-concentrated tryptic soy broth (TSB) and the microtitre plates were inoculated with 100 µL per well according to the manufacturer’s recommendation. These plates contained the biocides in 11 or 12 two-fold dilution steps: benzalkonium chloride (0.000008–0.016%), octenidine (0.000016–0.016%), chlorhexidine (0.000008–0.008%), and polyhexanide (0.000016–0.032%). The reference strain *Pseudomonas aeruginosa* ATCC^®^ 15442 served as quality control [34].

### 2.4. Macrorestricton Analysis with Subsequent Pulsed-Field Gel Electrophoresis

Macrorestricton analysis using the enzyme ApaI (New England Biolabs, Frankfurt, Germany) and subsequent pulsed-field gel electrophoresis (PFGE) were performed for a preliminary characterisation of the 99 *A. baumannii* isolates as previously published [35], with a minor modification: for restriction analysis with ApaI (30U), the plug slices were incubated overnight at 25 °C. A Lambda PFGE ladder (New England Biolabs, Frankfurt, Germany) with a size range from 48.5 to 1018 kb served as size marker. Electrophoresis was performed using the CHEF-DR III system (Bio-Rad Laboratories, Düsseldorf, Germany). Gels were stained with GelRed (Biotium, San Francisco, CA, USA) and scanned with the laboratory’s imaging system (BIO RAD Molecular Imager GelDoc^TM^ XR+ with Image Lab^TM^ Software, Düsseldorf, Germany). An isolate from diagnostics (141_Diagnostik) served as internal control on each gel. Cluster analysis concerning the percentage similarity was performed with BioNumerics software, version 7.6.3 (Applied Maths, bioMérieux). Similarities were calculated with the dice coefficient (optimization 1.5%, tolerance 1.5%) and the unweighted pair group method with arithmetic mean (UPGMA) [35]. Pulsotypes were defined at a threshold value of ≥80% (named alphabetically) and at a threshold value of ≥87% (additional numeric marking) [35,36].

### 2.5. Whole-Genome Sequencing

For whole-genome sequencing (WGS), 26 isolates were selected, including at least one isolate per PFGE pulsotype (cut off level of ≥80%). DNA was isolated using the Master Pure DNA Purification Kit for Blood Version II (Epicentre Biotechnologies) as published by the manufacturer. The libraries were prepared using the Nextera XT DNA Library Preparation Kit (Illumina Inc., San Diego, CA, USA) according to the manufacturer’s instructions. The 2 × 300 bp paired-end sequencing in 40-fold multiplexes was performed on the Illumina MiSeq platform (Illumina) with MiSeq Reagent Kit v3 (600-cycle) (Illumina). For sequence assembly, the Illumina reads were trimmed by Trim Galore v0.6.6 (RRID:SCR_011847) and quality checked by FastQC [37]. De novo assembling was carried out using Unicycler v0.4.9. [38]. Antimicrobial resistance genes were detected using ABRicate [39] with NCBI AMRFinderPlus [40], ResFinder [41], and CARD [42] databases. Plasmid replicons were searched for using ABRicate [39] applied to the PlasmidFinder 2.1 database (https://cge.food.dtu.dk/services/PlasmidFinder/ accessed on 21 February 2023). The databank PubMLST (https://pubmlst.org/ accessed on 21 February 2023 [43]) was used to confirm the species with ribosomal multilocus sequence typing (rMLST) [44] and to compare and identify sequence types (ST) using both the Pasteur [45] and the Oxford [46,47] scheme. New STs and new alleles were submitted to PubMLST [43]. The generated genomes were used for core genome multilocus sequence typing (cgMLST) with SeqSphere^+^ v7.5.5 (Ridom GmbH, Münster, Germany) [48]. This typing scheme is based on a core genome of 2390 alleles. However, the calculations for the minimum spanning tree presented here were done on the basis of only 1943 alleles as all missing values were excluded. Detected β-lactamases were compared with those listed in the Beta-Lactamase DataBase (www.bldb.eu accessed on 21 February 2023) [49]. Accession numbers and bioproject number are presented in the Data Availability section.

## 3. Results

### 3.1. Isolation

Ninety-nine *A. baumannii* isolates were collected during the study period. *A. baumannii* was detected in 79.7% (n = 94) of the 118 chick-box-papers. In two chick-box-papers, two morphologically different *A. baumannii* isolates were recovered. Two further *A. baumannii* isolates (2.4%) were found among the 82 boot swab samples tested from turkeys before slaughter. None of the 50 boot swab samples taken during the rearing period were positive for *A. baumannii* (Table 1). Taken together, 1.5% of the boot swab samples contained *A. baumannii.* The six unused chick-box-papers tested negative. *A. baumannii* was detected in one of the 217 swabs (0.5%) sent in for bacteriological diagnostics. The single positive pooled heart-lung-swab originated from a seven-week old turkey (isolate 141_Diagnostik). All of the 88 one- to six-day-old commercial turkey chicks were negative for *A. baumannii* in their liver and in their yolk sac (Table 1).

In total, there were 13 farms from which several *A. baumannii* isolates were detected (minimum two isolates, maximum five isolates). Only in one of them (farm 13) *A. baumannii* was detected in a chick-box-paper (isolate 16_W23.1) as well as in a boot swab sample before slaughter (isolate 98_E23.3) (Figure S1).

### 3.2. Antimicrobial Susceptibility Testing

The results of the antimicrobial susceptibility testing are displayed in Table 2. As there are no CLSI-approved veterinary-specific clinical breakpoints currently available for *A. baumannii*, human clinical breakpoints were applied. Using these interpretive criteria, all tested isolates were susceptible to imipenem and gentamicin. A high percentage of the tested isolates was susceptible to doxycycline (98%), trimethoprim/sulfamethoxazole (98%), and tetracycline (96%). Concerning cefotaxime, 31% of the isolates were classified as susceptible, 67% as intermediate, and 3% as resistant, despite the fact that the MICs of cefotaxime revealed a unimodal distribution with a mode MIC value of 16 mg/L. For ciprofloxacin, 83% of the isolates were susceptible and 17% were resistant. Bimodal MIC distributions, with two peaks representing a “susceptible” wildtype population and a non-wildtype population with acquired resistance properties, were seen for all the (fluoro)quinolones, including nalidixic acid, ciprofloxacin, enrofloxacin, and marbofloxacin. The same 17 isolates classified as ciprofloxacin-resistant also showed elevated MIC values for nalidixic acid as well as the veterinary fluoroquinolones enrofloxacin and marbofloxacin. All isolates were classified as intermediate to colistin. For the other tested antimicrobial agents there were no clinical breakpoints available. The MIC values were high especially for tiamulin, cefoperazone, and florfenicol, which is in accordance with the intrinsic resistance properties of *A. baumannii*. Bimodal MIC distributions were also seen for the tetracyclines, namely tetracycline and doxycycline, and also for trimethoprim/sulfamethoxazole.

### 3.3. Biocide Susceptibility Testing

The MIC values for the tested biocides all showed a unimodal distribution. They ranged as follows: benzalkonium chloride 0.0005–0.002%, octenidine 0.000125–0.002%, chlorhexidine 0.000125–0.008%, and polyhexanide 0.000125–0.008% (Table 3).

### 3.4. Macrorestricton Analysis with Subsequent Pulsed-Field Gel Electrophoresis

At the cut off level of ≥ 80%, there were 21 PFGE pulsotypes (A-U) containing up to 21 isolates (pulsotype P). At the cut off level ≥ 87%, there were 33 PFGE pulsotypes. These included up to 11 isolates per pulsotype and up to nine isolates with indistinguishable PFGE patterns (pulsotype B1), including isolates from different farms and different arrival dates (Figure S1).

### 3.5. Whole-Genome Sequencing

The whole-genome sequencing results are listed in Table 4. rMLST confirmed the assignment to the species *A. baumannii* with 100% support in all sequenced isolates.

Concerning the resistance genes, all sequenced isolates showed the presence of Ambler class D and Ambler class C β-lactamase genes (*bla*_OXA-51-like_ and *bla*_ADC_), which are intrinsic to *A. baumannii* [50,51,52,53]. Seventeen different *bla*_OXA_ β-lactamase variants were detected. Two isolates had *bla*_OXA-51_ β-lactamase genes and the other 24 isolates had *bla*_OXA-51-like_ β-lactamase genes, among which *bla*_OXA-64_ was by far the most prevalent with 38% (n = 10). All *bla*_OXA_ β-lactamase genes detected were confirmed and the deduced amino acid sequences confirmed their assignment (100% identity), except isolate 22_W33.1, which had a single amino acid difference (Met84Ile) to OXA-69 (99.8% identity). Eleven different *bla*_ADC_ β-lactamase gene variants were detected. Here, *bla*_ADC-26_ was the most common with 50% (n = 13), mostly co-located with *bla*_OXA-64_. In eight isolates, ADC β-lactamases were found, which exhibited less than 100% identity to known ADC variants. Four isolates exhibited one amino acid difference in the deduced protein sequences: isolate 54_W70.1 and isolate 29_W43.1 showed 99.7% identity to ADC-26 (Leu44Phe), respectively, isolate 31_W46.3 showed 99.7% identity to ADC-158 (Thr123Ala) and isolate 57_XXE4 showed 99.7% identity to ADC-192 (Gln2Arg). Two isolates had two amino acid differences: isolate 22_W33.1 showed 99.5% identity to ADC-159 (Glu118Lys and Ala270Thr) and isolate 98_E23.3 showed 99.5% identity to ADC-192 (Gln2Arg and Asp24Asn). Three amino acids differences were found in two isolates: isolate 32_W47.2 showed 99.2% identity to ADC-163 (Lys163Gln, Val197Ala, and Phe263Leu), and isolate 3_W5.2 showed 99.2% identity to two different ADC β-lactamases, namely ADC-158 (Thr112Lys, Pro216Ala, Arg274Lys) and ADC-274 (Ala99Gly, Pro216Ala, and Arg274Lys). The aminoglycoside nucleotidyltransferase gene *ant(3″)-IIa* was present in all isolates. Additional aminoglycoside O-phosphotransferase genes *aph(3″)-Ib* and *aph(6)-Id* were only identified in one isolate (35_W50.1). The *tet*(39) gene was found in two isolates, which were classified as tetracycline-resistant.

The *sul2* gene was detected in one of the two isolates, which showed resistance to trimethoprim/sulfamethoxazole. In the ten sequenced ciprofloxacin-resistant isolates, two gene mutations were detected in *gyrA* and *parC* genes resulting in the amino acid substitutions Ser81Leu (GyrA) and Ser84Leu or Ser84Phe (ParC), respectively. Isolate 71_W90.3, which showed an elevated MIC value for nalidixic acid but not for ciprofloxacin, had only a mutation in *gyrA,* which resulted in the amino acid substitution Ser81Leu (Table 4).

**Figure 1:**
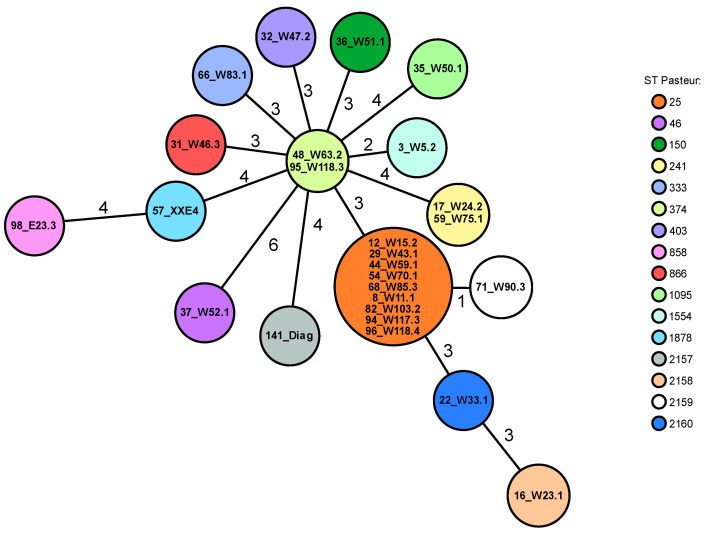
Minimum spanning tree, created with the Ridom SeqSphere+ software, showing the clonal relationship of 26 *A. baumannii* isolates based on Pasteur sequence types (ST). Each circle represents an allelic profile and the connecting lines display the number of different alleles between the distinct profiles. The individual isolate IDs are shown within the circles. The STs are indicated by colour as shown in the legend. The isolate 141_Diagnostik has been abbreviated as 141_Diag.

Multilocus sequence typing (MLST) analysis using the Pasteur scheme revealed 16 different STs (Table 4). Four STs (2157, 2158, 2159, and 2160) were newly described, and a new *fusA* allele (detected in isolate 71_W90.3 with the new ST2159), namely Pas_fusA-407, was newly added to the PubMLST database. By far the most commonly detected ST was ST25, comprising nine isolates (35%), followed by ST241 and ST374 with two isolates each (8%), respectively. The 12 remaining isolates all showed individual allelic profiles and different STs (Figure 1).

In the MLST analysis using the Oxford scheme, 18 different STs were present. Ten of these STs (2769, 2771, 2772, 2773, 2774, 2775, 2776, 2777, 2778, and 2779) were newly described, including three new alleles, which were added to the PubMLST database. ST1588 was the most common, including five isolates (19%), followed by ST229 including three isolates (12%), and ST1416 and ST2774 with two isolates each (8%) (Table 4).

**Figure 2:**
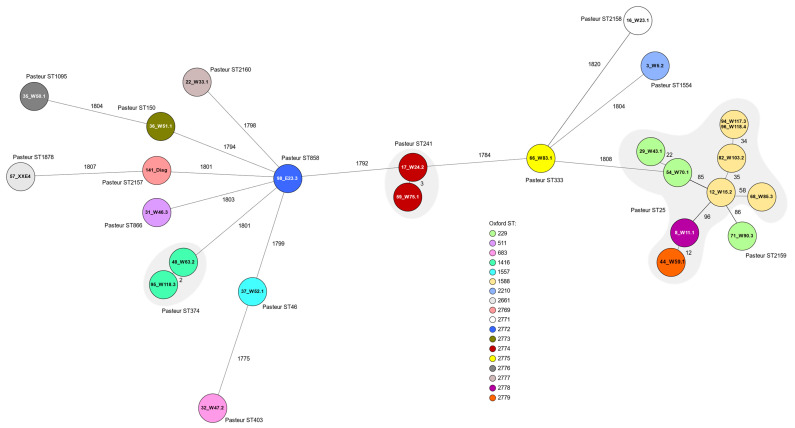
Minimum spanning tree showing the clonal relationship of 26 *A. baumannii* isolates based on a core genome multilocus sequence typing (cgMLST) analysis including 1943 alleles using the Ridom SeqSphere + software. Missing values were not included. Each coloured circle represents an allelic profile and the numbers adjacent to the connecting lines display the numbers of different alleles between the isolates. The individual isolate IDs are shown within the circles. The Oxford sequence types (ST) are indicated by colour as shown in the legend. The Pasteur STs are stated next to the circles, and if more than one isolate belongs to the same Pasteur ST, this is marked by grey clouds. The isolate 141_Diagnostik has been abbreviated as 141_Diag.

cgMLST using 1943 alleles for distance calculation showed a wide distribution of the 26 isolates tested. Most of these isolates showed a distinct allelic profile and were not closely related. They showed differences between 1775 and 1820 alleles. There was one cluster with ten related isolates (only up to 96 alleles apart). These ten isolates belonged to the Pasteur STs 25 and 2159 (new) and the Oxford STs 229, 1588, 2778 (new), and 2779 (new). The corresponding isolates all showed fluoroquinolone resistance. Otherwise, only two isolate pairs had closely related allelic profiles: isolates 48_W24.2 and 95_W75.1 with only two alleles difference, and isolates 17_W63.2 and 59_W118.3 with three alleles difference (Figure 2).

## 4. Discussion

In the chick-box/meconium samples from one-day-old turkey chicks, there was a very high presence of *A. baumannii*. Overall, 79.9% of the chick-box-papers contained *A. baumannii* isolates. Intriguingly, the highest detection rate in birds (25% from n = 661) up till now was also found in white stork nestlings. Other findings in goslings and chickens seem to have also been especially prevalent in younger birds [18]. Interestingly, the detection rate of *A. baumannii* found in boot swab samples (n = 132) taken during rearing and before slaughter was low, with only 1.5%. Our results, therefore, highlight that the presence of *A. baumannii* in samples from poultry can vary considerably with the age of the birds and is transient. In another study, for example, *A. baumannii* was not isolated in bioaerosols from a housing with 7-week-old turkeys [54]. The detection of only one *A. baumannii* isolate in 217 lung-heart swabs (0.5%) and in none of the yolk sac and liver samples from one-to six-day-old turkey chicks during diagnostics, additionally points towards a generally low presence of *A. baumannii* in fattening turkeys. The data, therefore, suggest, as in wild birds [24], that there is no evidence for a general preference of *A. baumannii* for avian hosts. With regard to the diagnostic samples in this study, there was also no evidence of *A. baumannii* playing a role in diseased turkeys.

The preliminary characterisation via PFGE revealed that in total, the *A. baumannii* isolates found in this study were very heterogenous, forming 21 pulsotypes at a cut off level of *≥* 80% and 33 pulsotypes at a cut off level of *≥* 87%, comprising between one and eleven isolates. Core genome MLST highlighted the diverse population of *A. baumannii* isolates found in this study. However, as anticipated, the PFGE results did not completely correspond with the core genome MLST data of the 26 isolates subjected to WGS. Due to the very heterogenous isolates, which were not closely related, an environmental origin as discussed in the studies on storks [18] and cattle [16] seems likely. The source of the *A. baumannii* isolates is not clear. To investigate possible reservoirs in future studies, the environment of one-day-old chicks, i.e., hatcheries and transport vehicles, should be analysed. In other studies, *A. baumannii* isolates have been found in the air of a duck hatchery [27] and non-sterile water (which is used for humidity regulation during the brood), which has been suggested to be a possible source of contamination in hatcheries [26]. Moreover, feather down has also been considered as a potential carrier [26,55]. In general, *Acinetobacter* spp. are widespread environmental microorganisms [56] and *A. baumannii*, for example, can be detected in soil [57,58].

Antimicrobial susceptibility testing revealed that the majority of isolates were susceptible to a wide range of antimicrobial agents, which is comparable with the results obtained from cattle and white storks as well [16,18]. Multidrug resistance properties were not detected. In addition to the species-specific intrinsic resistance properties, only two isolates showed acquired resistance to two different classes of antimicrobial agents, namely (fluoro)quinolones and tetracyclines. The highest resistance rates were detected for ciprofloxacin. The MIC values of the other tested quinolones, for which no clinical breakpoints exist, confirmed this finding. The detected mutations in *gyrA* and *parC* genes, respectively, are linked to fluoroquinolone selection of resistance, which suggests that the isolates have been circulating in an environment where fluoroquinolones have been used [59]. Interestingly, the isolate showing only one mutation in the *gyrA* gene and none in the *parC* gene revealed only an elevated MIC value for nalidixic acid, but not one for ciprofloxacin. The tetracycline resistance in two isolates could be attributed to the *tet*(39) gene, which encodes an active efflux mechanism and has been described in *Acinetobacter* spp. found frequently in the aquatic environment [60,61]. As *A. baumannii* is intrinsically resistant to trimethoprim, resistance to trimethoprim/sulfamethoxazole could only be attributed to the gene *sul2*, which confers resistance to sulfonamides [62] in one isolate. The cause of the resistance in the other trimethoprim/sulfamethoxazole-resistant isolate (36_W51.1) was not resolved as no further *sul* genes nor mutations in the genes *folA* and *folP* could be detected. The aminoglycoside nucleotidyltransferase gene *ant(3″)-IIa* was present in all isolates as described in other studies [63,64]. Phosphotransferase *aph(3″)-Ib* and *aph(6)-Id*, which mediate streptomycin resistance, were both detected in isolate 35_W50.1 (streptomycin MIC value 64 mg/L). The streptomycin MIC values of the remaining 25 isolates, which did not harbour these two resistance genes, varied between 4 mg/L and ≥128 mg/L. One of the three isolates resistant to cefotaxime (isolate 17_W24.2) was examined by WGS and no additional beta-lactamase gene, except the intrinsic ones, could be identified. The classification of the isolates as cefotaxime-resistant may be due to the unimodal MIC distribution of the tested *A. baumannii* isolates around the clinical breakpoint. In other studies, cefotaxime resistance has been described in association with the production of the CTX-M-2 extended spectrum class A β-lactamase [65,66]. In general, *bla*_OXA_ genes are found on both chromosome and plasmids, and it might also be possible that more than one copy of *bla*_OXA_ is on the chromosome [67]. The isolates tested in this study showed a diverse selection of intrinsic *bla*_OXA_ β-lactamase genes. It is important to say that we did not detect acquired β-lactamase genes, such as *bla*_OXA-23_ or *bla*_OXA-58_, which are associated with carbapenem resistance [2] in any of the 26 sequenced isolates. The gene *bla*_OXA-64_, which was the most frequently detected *bla*_OXA_ gene in this study, has previously been found in feather down and dust from turkey and goose hatcheries [18]. It correlates with the Pasteur ST25 [68], except in the case of isolate 71_W90.3, which interestingly showed a new Pasteur type, ST2159 (with a new *fusA* allele), but a known Oxford type ST229. Some other *bla*_OXA_ β-lactamase genes found in our study have been detected in samples from other avian species as well, i.e., *bla*_OXA-51_ (white stork choana), *bla*_OXA-68_ (chicken choana, feather down and dust from a chicken hatchery), *bla*_OXA-104_ (white stork choana), *bla*_OXA-208_ (white stork pellet), and *bla*_OXA-385_ (1-day-old chicken choana) [18]. Interestingly, Wilharm et al. could assign two chicken samples with *bla*_OXA-68_ (Pasteur ST23) to the international clone 8 (IC8) [18], which includes outbreak strains in human medicine (Pasteur STs 10 and 157) [69]. Only one isolate (16_W23.1) in this study had *bla*_OXA-68_. This isolate showed new Pasteur and Oxford STs. Furthermore, a variety of different *bla*_ADC_ β-lactamase genes were found. The most common, *bla*_ADC-26_, was mostly present in isolates that also carried *bla*_OXA64_, except for isolate 37_W52.1, which was characterised by *bla*_OXA-104_, Pasteur ST46 and Oxford ST1557, and the isolates 48_W63.2 and 95_W118.3, characterised by *bla*_OXA-259_, Pasteur ST374 and Oxford ST1416.

Biocide susceptibility testing revealed that the MIC values for the four biocides were all distributed unimodally. There is not much data concerning biocide susceptibility available for comparison. In one study, 14 *A. baumannii* isolates from dogs and cats were examined using the same protocol [70]. Interestingly, the MIC ranges were generally wider in our study presented here, often starting at lower dilution steps. However, it has to be kept in mind that this could be due to the number of isolates tested (n = 99 vs. n = 14).

MLST analysis revealed many different STs, which highlights the heterogenous nature of the isolates. Pasteur ST25, which was detected in 35% of the sequenced isolates, was most prevalent. In humans, ST25 is a successful lineage, which can lead to epidemics, has spread worldwide, and belongs to the international clone 7 (IC7) [68,69,71,72,73,74,75]. All ST25 isolates described here carry *bla*_OXA-64_ and were resistant to ciprofloxacin, which corresponds to the results of other studies [68,71]. Only one of the ten ciprofloxacin-resistant isolates examined by WGS (isolate 66_W83.1) belonged to Pasteur ST333, which was first described in China [76]. Two of our isolates were susceptible to ciprofloxacin and belonged to Pasteur ST374, which, according to the PubMLST database, occurs worldwide. The ST374 lineage is grouped into the clonal complex CC3 belonging to the international clone IC3 [77]. Two further isolates belonged to Pasteur ST241, which has been detected in human samples across the world according to PubMLST database. In Germany, it has been found in a cattle faecal sample [16] and in milk powder [63]. Interestingly, our two ST241 isolates, which were only three alleles apart in cgMLST, had a new Oxford sequence type ST2774, harboured *bla*_OXA-91_ and *bla*_ADC-52_, and showed an elevated cefquinome MIC of ≥64 mg/L. None of the Pasteur STs in this study corresponded to those found in chicken and turkey meat in Switzerland [29]. More Oxford STs (n = 18) were found in this study in comparison to the Pasteur STs (n = 16). The Pasteur ST25 comprised four different Oxford STs (ST1588, ST229, ST2779 (new), and ST2778 (new)).

In general, it can be concluded for commercial turkeys, as it has been for cattle [16] and storks [18], that the population of *A. baumannii* is highly diverse and still susceptible to many antimicrobial agents. The overall occurrence of *A. baumannii* in samples from commercially reared turkeys seems to be very low. Only chick-box-papers were found to harbour large numbers of *A. baumannii* isolates. Although *Acinetobacter* isolates have been obtained from rhizospheric soil, tomato, and cauliflower roots [78], a transfer from these sources to the animals investigated in this study can be excluded as the turkey chicks/turkeys did not have contact to these matrices. Thus, the possible origin of the *A. baumannii* isolates found in this study remains to be elucidated and will be a subject for further investigation.

## Figures and Tables

**Table 1:** Overview of all study samples and the occurrence of *A. baumannii*.

	Samples	No.	No. Positive	Detection Rate (%)	No. of Isolates
	chick-box-papers (meconium samples)	118	94	79.7	96 *
	boot swab samples during rearing	50	0	0	0
	boot swab samples before slaughter	82	2	2.4	2
	lung-heart swabs (diagnostics)	217	1	0.5	1
	liver (diagnostics)	88 ^#^	0	0	0
	yolk sac (diagnostics)	88 ^#^	0	0	0
total		643	97	15.1	99

* Two morphologically different *A. baumannii* isolates were recovered from each of two chick-box papers; ^#^ Liver and yolk sac were tested separately in 88 chicks.

**Table 2:** Minimal inhibitory concentration (MIC) distributions of 99 *A. baumannii* isolates tested on 18 antimicrobial agents.

Antimicrobial Agent	No. of Isolates for Which the MIC (mg/L) Is ^a^:																		MIC_50_ (mg/L)	MIC_90_ (mg/L)
	0.008	0.015	0.03	0.06	0.12	0.25	0.5	1	2	4	8	16	32	64	128	256	512	1024		
Colistin							17	76	6										1	1
Streptomycin										12	22	20	23	18	4				16	64
Neomycin						1	10	53	33	2									1	2
Trimethoprim/ Sulfamethoxazole (1:19) ^**b**^				1	37	44	15				1	1							0.25	0.5
Gentamicin					1	6	49	40	3										0.5	1
Nalidixic Acid								1	19	38	22	1		1		17			4	≥256
Ciprofloxacin				3	19	33	24	3			1	4	12						0.25	≥32
Enrofloxacin		4	13	50	12	3			3	6	7	1							0.06	4
Marbofloxacin			2	41	35	3	1		2	10	4	1							0.12	4
Tetracycline							3	27	51	14	2			1		1			2	4
Doxycycline				12	45	32	8				1	1							0.12	0.5
Florfenicol													1	20	61	17			128	256
Imipenem					37	61	1												0.25	0.25
Ceftiofur										1	3	66	28	1					16	32
Cefquinome						1		11	22	44	14	1	4	2					4	8
Cefotaxime									1	4	26	45	20	3					16	32
Cefoperazone												1	31	67					≥64	≥64
Tiamulin															99				≥128	≥128

The black areas represent concentration steps not included in the test panels. Grey shadings mark the categories according to CLSI (dark grey for resistant, middle grey for intermediate, and light grey for susceptible). **^a^** MIC values equal to or lower than the lowest concentration tested are given as the lowest concentration tested; MIC values equal to or higher than the highest concentration tested are given as one concentration step above the highest tested concentration (white number on black background). **^b^** The MIC values of trimethoprim/sulfamethoxazole (1:19) are expressed as the MIC values of trimethoprim.

**Table 3:** Distribution of the MIC values for 99 *A. baumannii* isolates tested for four biocides.

Biocide Agent	No. of Isolates for Which the MIC (%) Is:						
	0.000125	0.00025	0.0005	0.001	0.002	0.004	0.008
Benzalkonium chloride	-	-	29	51	19	-	-
Octenidine	10	50	34	4	1	-	-
Chlorhexidine	10	8	9	24	34	13	1
Polyhexanide	3	12	32	25	19	5	3

**Table 4:** Overview of the results of the 26 *A. baumannii* isolates which were investigated by whole-genome sequencing.

ID	PFGE	Pasteur ST ^1^	Oxford ST ^1^	Resistance Phenotype ^2^	*bla*_OXA_^3^	*bla*_ADC_^3^	*ant(3″)-IIa*	*aph(3″)-Ib aph(6)-Id*	*sul* *2*	*tet*(39)	GyrA	ParC	Accession Number
68_W85.3	A1	25	1588	NAL, CIP	64	26	x ^4^				Ser81Leu	Ser84Leu	JAPQZB010000000
54_W70.1	A2	25	229	NAL, CIP	64	26 (99.7%)	x				Ser81Leu	Ser84Leu	JAPQYW010000000
29_W43.1	A3	25	229	NAL, CIP	64	26 (99.7%)	x				Ser81Leu	Ser84Leu	JAPQYV010000000
17_W24.2	B1	241	2774	FOT	91	52	x						JAPQYR010000000
82_W103.2	C	25	1588	NAL, CIP, (TET)	64	26	x				Ser81Leu	Ser84Leu	JAPQZA010000000
94_W117.3	D2	25	1588	NAL, CIP	64	26	x				Ser81Leu	Ser84Leu	JAPQYZ010000000
3_W5.2	E	1554	2210		424	158/274 (99.2%)	x						JAPQYH010000000
48_W63.2	F	374	1416		259	26	x						JAPQYL010000000
71_W90.3	G	2159	229	NAL	64	26	x				Ser81Leu		JAPQZC010000000
44_W59.1	H	25	2779	NAL, CIP, TET, DOX	64	26	x			x	Ser81Leu	Ser84Leu	JAPQYU010000000
8_W11.1	H	25	2778	NAL, CIP, TET, (DOX)	64	26	x			x	Ser81Leu	Ser84Leu	JAPQYT010000000
59_W75.1	I	241	2774		91	52	x						JAPQYQ010000000
12_W15.2	J	25	1588	NAL, CIP	64	26	x				Ser81Leu	Ser84Phe	JAPQYY010000000
96_W118.4	K1	25	1588	NAL, CIP	64	26	x				Ser81Leu	Ser84Leu	JAPQYX010000000
35_W50.1	L	1095	2776	SXT	208	249	x	x	x				JAPQYM010000000
66_W83.1	M1	333	2775	NAL, CIP	111	179	x				Ser81Leu	Ser84Leu	JAPQYO010000000
22_W33.1	M2	2160	2777		69 (99.8%)	159 (99.5%)	x						JAPQYG010000000
32_W47.2	N	403	683		263	163 (99.2%)	x						JAPQYJ010000000
141_Diag *	O	2157	2769		51	165	x						JAPQZE010000000
57_XXE4	O	1878	2661		863	192 (99.7%)	x						JAPQYF010000000
16_W23.1	P1	2158	2771		68	76	x						JAPQYS010000000
36_W51.1	Q2	150	2773	SXT	121	163	x						JAPQYN010000000
98_E23.3	R	858	2772		51	192 (99.5%)	x						JAPQZD010000000
37_W52.1	S1	46	1557	(TET)	104	26	x						JAPQYP010000000
31_W46.3	T	866	511		385	158 (99.7%)	x						JAPQYI010000000
95_W118.3	U1	374	1416		259	26	x						JAPQYK010000000

^1^ Numbers highlighted in light grey represent new STs; numbers highlighted in dark grey represent new STs including new alleles; ^2^ NAL: nalidixic acid; CIP: ciprofloxacin; FOT: cefotaxime; TET: tetracycline; DOX: doxycycline; SXT: trimethoprim/sulfamethoxazole; ^3^ 100 percent identity on amino acid level (unless otherwise indicated); ^4^ x = present; * 141_Diag is the abbreviation for 141_Diagnostik.

## Data Availability

All data presented in this study are available in the text, figures and tables of the main article and in the supplementary material. Whole-genome sequences of the *A. baumannii* isolates included in this study are available at DDBJ/ENA/GenBank under the bioprojekt number PRJNA904545 and the accession numbers JAPQZE010000000, JAPQZD010000000, JAPQZC010000000, JAPQZB010000000, JAPQZA010000000, JAPQYZ010000000, JAPQYY010000000, JAPQYX010000000, JAPQYW010000000, JAPQYV010000000, JAPQYU010000000, JAPQYT010000000, JAPQYS010000000, JAPQYR010000000, JAPQYQ010000000, JAPQYP010000000, JAPQYO010000000, JAPQYN010000000, JAPQYM010000000, JAPQYL010000000, JAPQYK010000000, JAPQYJ010000000, JAPQYI010000000, JAPQYH010000000, JAPQYG010000000 and JAPQYF010000000.

## Supplementary Materials

The following supporting information can be downloaded at: https://www.mdpi.com/article/10.3390/microorganisms11030759/s1, Figure S1: Pulsed-field gel electrophoresis profiles from 99 *A. baumannii* isolates.
