# Supplementary material for: Acinetobacter baumannii from Samples of Commercially Reared Turkeys: Genomic Relationships, Antimicrobial and Biocide Susceptibility
Source: Microorganisms. 2023 Mar 16;11(3):759. doi: 10.3390/microorganisms11030759 (PMC10052703; doi:10.3390/microorganisms11030759)
Supplement: Supplementary file 1 [file microorganisms-11-00759-s001.zip › microorganisms-2230265-supplementary.pdf]

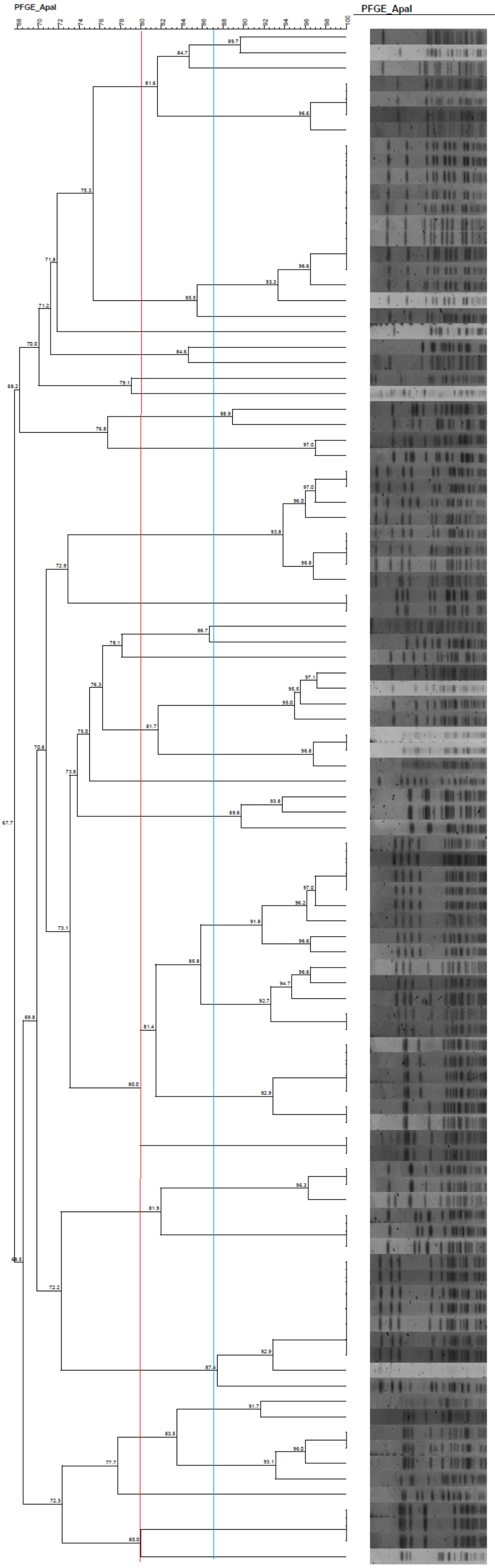

Isolate ID	Date	Comments	
15_W18.4	23.05.2019		A1
68_W85.3	04.11.2019		
54_W70.1	19.10.2019		A2
6_W9.3	08.05.2019		A3
29_W43.1	17.07.2019		
52_W67.1	18.10.2019	Farm 4	
88_W109.4	03.12.2019		
39_W54.1	30.09.2019	Farm 12	B1
40_W55.1	30.09.2019	Farm 12	
49_W64.1	25.09.2019		
17_W24.1	18.06.2019		
26_W40.4	11.07.2019	Farm 10	
53_W68.1	18.10.2019		
60_W76.1	23.10.2019	Farm 5	
87_W108.2	05.12.2019		
78_W100.2	13.11.2019	Farm 6	
70_W87.1	06.11.2019		
55_W31.1	24.06.2019		
73_W93.1	19.11.2019		B2
82_W103.2	25.11.2019	Farm 11	C
43_W58.1	07.10.2019	Farm 10	D1
94_W117.3	13.12.2019		D2
3_W5.2	17.04.2019		E
48_W63.2	14.10.2019		F
9_W12.2	16.05.2019		G
71_W90.3	12.11.2019		
8_W11.1	16.05.2019		H
44_W59.1	07.10.2019	Farm 8	
42_W57.1	07.10.2019	Farm 10	I
72_W92.1	13.11.2019	Farm 9	
19_W26.3	20.06.2019		
25_W36.1	08.07.2019	Farm 8	
47_W61.1	09.10.2019		
24_W35.1	08.07.2019	Farm 8	
59_W75.1	23.10.2019		
4_W6.1	06.05.2019		
10_W13.4	18.05.2019		J
12_W15.2	24.05.2019		
96_W118.4	13.12.2019		K1
67_W84.3	31.10.2019	Farm 2	K2
35_W50.1	02.10.2019		L
30_W44.1	27.09.2019		M1
69_W86.1	07.11.2019		
33_W48.1	26.09.2019	Farm 3	
66_W83.1	31.10.2019		
18_W25.3	17.06.2019		M2
21_W32.2	04.07.2019		
22_W33.1	04.07.2019	Farm 6	
32_W47.2	25.09.2019		N
57_XXE4	23.07.2019	boot-swab	O
58_W74.2	23.10.2019		
141_Diagnostik	n.a.	lung-heart-swab	
16_W23.1	14.06.2019	Farm 13	P1
38_W53.3	02.10.2019		
76_W97.1	22.11.2019		
79_W101.3	22.11.2019		
77_W98.1	19.11.2019	Farm 7	
91_W113.2	06.12.2019		
13_W16.2	23.05.2019	Farm 7	
34_W49.1	01.10.2019		
62_W79.1	26.10.2019	Farm 1	P2
97_W119.3	12.12.2019		
85_W106.2	26.11.2019	Farm 5	
86_W107.2	28.11.2019		
90_W112.2	06.12.2019		
50_W65.1	14.10.2019		P3
84_W105.1	26.11.2019		
74_W94.1	19.11.2019		
75_W95.2	25.11.2019		
14_W17.3	23.05.2019	Farm 11	
51_W66.1	14.10.2019		
92_W115.3	06.12.2019		P4
89_W111.3	04.12.2019		
23_W34.1	11.07.2019		Q1
27_W41.2	15.07.2019	Farm 4	
61_W77.3	23.10.2019		
1_W3.1	24.04.2019	Farm 1	Q2
36_W51.1	28.09.2019	Farm 1	
63_W80.1	26.10.2019	Farm 1	
5_W7.1	08.05.2019		R
7_W10.2	08.05.2019	Farm 9	
41_W56.1	14.09.2019		
28_W42.1	12.07.2019		
56_W72.1	17.10.2019		
93_W116.2	11.12.2019		
46_W61.2	09.10.2019		
98_E23.3	14.06.2019*	Farm 13, boot swab	
45_W60.1	08.10.2019		
20_W27.1	20.06.2019		S1
37_W52.1	02.10.2019		
11_W14.2	24.05.2019	Farm 3	S2
64_W81.1	05.11.2019	Farm 11	
65_W82.1	05.11.2019	Farm 11	
2_W4.1	24.04.2019	Farm 2	
31_W46.4	25.09.2019		T
81_W103.1	25.11.2019	Farm 11	U1
83_W104.2	25.11.2019	Farm 11	
95_W118.3	13.12.2019		
80_W102.1	22.11.2019		U2

Figure S1. Pulsed-field gel electrophoresis profiles from 99 *A. baumannii* isolates. Similarity calculation was done by the BioNumerics software, with the dice coefficient and the Unweighted-Pair Group Method with Arithmetic Mean. Vertical lines indicate the threshold value ≥80% (red) and the threshold value ≥87% (blue). The dates given are the days on which the 1-day-old turkey chicks arrived at the production sites (n.a. = not available). All isolates were isolated from chick-box-papers, except those stated differently under comments. Farms, from which several samples were examined, are listed under comments. Horizontal lines indicate the pulsotypes, which are named in bold at the far right.
